# Feasibility of REBOA (Resuscitative Endovascular Balloon Occlusion of the Aorta) Implementation in HEMS (Helicopter Emergency Medical Service) Units in Castilla-La Mancha, Spain

**DOI:** 10.3390/nursrep16030085

**Published:** 2026-02-28

**Authors:** Antonio Martínez García, Iván Ortega-Deballon, Juan Manuel López-Reina Roldán, Andreu Martínez Hernández, Martín Torralba Melero, Rubén Quintero Mínguez

**Affiliations:** 1 Department of Nursing and Physiotherapy, Faculty of Medicine and Health Sciences, Universidad de Alcalá, 28805 Madrid, Spain; antoniomartinezenfer@gmail.com (A.M.G.); ivan.ortega@uah.es (I.O.-D.); 2 GUETS (Gerencia de Urgencias, Emergencias y Transporte Sanitario), SESCAM, 45071 Castilla la Mancha, Spain; 3 HEMS-112 Former, SERCAM, 28752 Madrid, Spain; 4 Department of Medicine, Jaume I University, 12070 Castellón, Spain; andmarti@uji.es; 5 Department of General Surgery, Castellón University General Hospital, 12004 Castellón, Spain; 6 HEMS, AVINCIS, 02007 Albacete, Spain; 7 Department of Intensive Care, Albacete University Hospital, 02006 Albacete, Spain

**Keywords:** balloon occlusion, helicopter medical service, aortic occlusion

## Abstract

**Introduction:** Currently, REBOA (Resuscitative Endovascular Balloon Occlusion of the Aorta) is an emerging technique for resuscitation in patients presenting severe pathology in hemodynamic shock refractory to conventional treatments. The REBOA technique consists of inserting a balloon through the femoral artery to temporarily occlude the aorta and thus control massive bleeding and improve perfusion of vital organs in critical situations such as hemorrhagic shock. Although it is not a definitive technique, its use buys time before the implementation of a definitive treatment when possible. This makes REBOA an ideal technique for the philosophy of out-of-hospital emergency services and more particularly in the HEMS (Helicopter Emergency Medical Service) environment. On the other hand, REBOA has been postulated as one of the basic pillars in the resuscitation of severe trauma patients with hemorrhagic shock and of the doctrine of damage-control resuscitation in non-compressible torso and lower limb hemorrhage. **Objective:** To evaluate the potential feasibility of REBOA implementation in patients attended by HEMS teams in Castilla-La Mancha, Spain. **Method:** A retrospective observational study was conducted analyzing medical and nursing reports from HEMS units between 1 January and 31 December 2023. A statistical study of the variables collected was carried out using statistical techniques appropriate to the pre-specified study variables. A descriptive analysis of the population was performed. Frequency results are expressed in absolute terms, as percentages and confidence intervals. Continuous variables are expressed as mean (SD) and median (range) according to normality test (Kolmogorov–Smirnov test). For the study of the relationship between the different variables, Chi-square or Analysis of Variance is used if they are parametric. Descriptive and inferential statistics were performed using SPSS v24. **Results:** A total of 103 patients (72.81% men, mean age 57.7 years) were identified as potential REBOA candidates. On arrival of the emergency services the mean SI (shock index) of the patients was 1.36 (SD +/− 0.380). On arrival at the hospital, the mean SI was 1.25 (SD +/− 0.601). Of the series, 57 (55.33%) patients suffered cardiorespiratory arrest (CRA) at some point during pre-hospital care. Of the total number of patients, 38 were patients presenting severe trauma criteria (characterized by life-threatening injuries, with RTS score ≤ 11, shock index > 0.9, or ISS ≥ 16, indicating severe physiological or anatomical alterations), of which 26 (68.4%) did not go into CRA, while 12 (31.6%) did. Of the total number of patients, 65 (63.1%) did not meet severe trauma criteria, but did present medical criteria for REBOA placement, of which 55 (53.4%) were patients who at some point during attendance presented CRA. Although the shock index showed a slight decrease after healthcare without statistical significance or relevant correlation, a highly significant association was observed between severe trauma and cardiorespiratory arrest (*p* < 0.001). **Conclusions**: It could be affirmed that it may have been feasible to implement REBOA in 4.47% (103) of the patients attended by the HEMS healthcare team of Castilla-La Mancha. This could help to reduce the morbimortality and mortality of critical patients in medical helicopters. More studies are needed to corroborate this assertion.

## 1. Introduction

Spain’s Helicopter Emergency Medical Services (HEMSs) are run by autonomous communities, yielding heterogeneous program models, aircraft, cabin layouts, and clinical capabilities. Given Spain’s geography and population dispersion, HEMSs provide indispensable access to time-critical care. While there is no HEMS-specific health regulation, civil aeronautical requirements (e.g., Royal Decree 279/2007) [1] and general health law govern air operations; by contrast, road medical transport is regulated by Royal Decree 836/2012 [2], which defines technical specifications, equipment, and staffing for ground ALS resources.

HEMS activity began with military inter-island transfers in 1955 and expanded in 1963 through the General Directorate of Traffic and the Red Cross to serve severely injured trauma patients [3]. Today, multiple public agencies operate medicalized helicopter bases, including some with night capability. Common platforms—Bell 415, EC135, EC145, AW109, and AW139—differ in performance and cabin configuration, complicating standardization of advanced procedures during flight. Severe trauma constitutes a substantial share of HEMS caseloads, and available evidence supports the safety of air transport for these patients [4,5,6,7].

Resuscitative Endovascular Balloon Occlusion of the Aorta (REBOA) is a temporizing resuscitative strategy for profound shock—particularly non-compressible torso hemorrhage—by endoluminal balloon occlusion to raise afterload, maintain arterial pressure, and secure a narrow window for definitive hemorrhage control [8,9]. In Spain, REBOA is predominantly deployed at tertiary hospitals upon arrival of patients with refractory hemodynamic instability; pre-hospital use is emerging in selected HEMS programs. Within damage-control resuscitation, REBOA is widely cited as a key adjunct for hemorrhagic shock due to non-compressible bleeding [10,11,12,13].

According to the 2021 European Resuscitation Council (ERC) guidelines [14,15], REBOA was recommended as an adjunct during traumatic cardiopulmonary resuscitation (tCPR). However, the updated 2025 ERC guidelines no longer recommend its use in traumatic cardiac arrest. This change reflects the evolution of scientific evidence, and is included here solely to contextualize current recommendations [16,17,18,19].

Spain has implemented trauma systems with protocolized pathways [11] (e.g., trauma codes) and regionalized referral to expedite arrival to specialized trauma ICUs and reduce morbidity and mortality. Nevertheless, geographic and demographic dispersion still produces prolonged transport times from rural or remote settings and frequent events far from specialized resources, reinforcing the role of ALS-equipped HEMS to shorten delays and promote equitable access [20,21,22,23,24,25,26].

In this context, feasibility is understood as the capacity of the severe trauma population attended by HEMS in Castilla-La Mancha to meet predefined REBOA inclusion and exclusion criteria. The primary objective is to determine whether this population presents sufficient feasibility—defined as the proportion of HEMS trauma patients who meet REBOA criteria—to support future REBOA implementation within the regional HEMS system. Secondary objectives are to describe the profile of these patients and to contextualize feasibility strictly in relation to eligibility and case volume, aligned with healthcare-equity considerations.

## 2. Materials and Methods

### 2.1. Study Design

A retrospective document-based study was conducted using official pre-hospital care reports generated by HEMS teams across Castilla-La Mancha between 1 January and 31 December 2023. The aim was to comprehensively characterize pre-hospital management and identify potential candidates for REBOA implementation. A standardized data extraction tool was designed using the clinical documentation entered by physicians and nurses during missions.

The objective variables collected are as follows.

Collected variables were organized to increase methodological clarity. Demographic data (sex and age), operational times (activation, arrival at the scene, patient contact, transfer times, and distance), vital signs (blood pressure, heart rate, respiratory rate, capnography, oxygen saturation, and glucose), and all performed interventions were documented, including vasoactive drugs, transfusion of blood products, trauma location, ECO-FAST findings, and estimated bleeding volume. Laboratory parameters and trauma severity scores (Glasgow Coma Scale (GWCS), Shock Index (SI), Modified Shock Index (MSI), Trauma Score Revised (TSR), AIS Scale (AIS—Abbreviated Injury Scale by trauma localization), ISS Scale (ISS—Injury Severity Score), NISS Scale (NISS—New Injury Severity Score)) were also recorded.

Additionally, CPR-related variables were included as follows: BLS initiation time, bystander CPR, ACLS initiation time, and total duration of resuscitation.

### 2.2. Review Process

Two independent investigators evaluated all reports between June and December 2024. Each reviewer conducted an individual systematic assessment, later merging findings into a consolidated dataset. Disagreements were resolved through consensus to ensure methodological rigor.

**Criteria for selection of care reports.**

All patients meeting the following characteristics will be included.

- Chronology: from 1 January 2023 to 31 December 2023.
- Medical helicopters of Castilla-La Mancha: Giant 1 (Albacete), Giant 2 (Ciudad Real), Giant 3 (Cuenca), Giant 4 (Toledo).
- Warnings in which advance notice was given by trauma code.
- Reports with Code ICD (International Classification of Diseases) 9 URGENCIAS GUETS (*Gerencia de Urgencias, Emergencias y Transporte Sanitario*, which is the Pre-hospital Care Service in Castilla-La Mancha) (Table 1).

**Exclusion criteria for report selection.**

Patients who do not comply with the inclusion data will be excluded, in addition to those care reports that for some reason we cannot extract the described variables or it is impossible to read them critically.

**Criteria for inclusion and exclusion of data from reports with patients who are candidates for REBOA:**

Considering the most current scientific evidence to date for this project, the inclusion and exclusion criteria for data from reports with patient candidates for REBOA have been selected.

**Non-traumatic CPR patients:**

For patients who have suffered non-traumatic cardiac arrest, we have selected the inclusion and exclusion criteria as specified in the following document [27,28]. The REBOARREST Trial Protocol, Version 1.3, 14 June 2021. Universal Trial Number: U1111-1253-0322:

The person must meet all the following criteria to be included in the study:

- Estimated age is between 18 and 80 years.
- Out-of-hospital cardiac arrest.
- Cardiac arrest of non-traumatic origin.
- Less than 10 min from the onset of arrest to the start of basic or advanced life support.
- Advanced life support is established and can be continued.
- Patients who experience cardiac arrest while being cared for by ambulance personnel or the HEMS emergency medical team.

Meeting only one of the following criteria excludes the person from being selected for non-traumatic CPR patients:

- Traumatic cardiac arrest, including strangulation, electrocution, and patients rescued from avalanches.
- Accidental hypothermia with temperature < 32 °C.
- Suspected cerebral hemorrhage as the cause of cardiac arrest.
- Suspected non-traumatic hemorrhage as the cause of cardiac arrest.
- Pregnancy, evident or suspected.
- Other factors determined by the treating team (environmental, safety or other factors).

**Traumatic CRA patients:**

In patients who have suffered cardiac arrest episodes of traumatic origin, the inclusion and exclusion criteria have been selected considering the algorithms and recommendations of the ERC 2021 [15]:

1. Traumatic cardiac arrest with suspected non-compressible hemorrhage in the abdomen or pelvis.
2. Absence of other immediate treatable causes (such as tension pneumothorax or cardiac tamponade).
3. Femoral vascular access is possible and rapid.

**Patients with refractory non-compressible hemorrhagic shock:**

**Inclusion criteria [13]**

1. Massive hemorrhage with persistent hemodynamic instability, defined as refractory shock, and including the following.
  - Systolic blood pressure < 90 mmHg despite resuscitation with fluids and/or blood products.
  - Shock index > 0.9 despite resuscitation with fluids and/or blood products.
2. Need for temporary bleeding control while preparing for definitive intervention.
3. Femoral vascular access is possible without anatomical contraindications.

**Exclusion criteria**

1. Hemorrhage controlled by conventional measures.
2. Anatomical or technical contraindications:
  - Aortic aneurysm, aortic dissection, femoral thrombosis, or impossible vascular access.
3. Bleeding above the diaphragm (upper esophageal or nasopharyngeal):
  - REBOA is not effective in these cases, as aortic occlusion does not control bleeds above zone 1.
4. Prolonged time from onset of bleeding without intervention (>6 h).
5. Patient with terminal prognosis or anticipated decisions not to resuscitate (DNR).

### 2.3. Statistical Analysis

Descriptive and inferential techniques were applied. Continuous variables were expressed as mean (SD) or median (range). Categorical variables were summarized as frequencies and percentages. Group comparisons used Chi-square, ANOVA, or nonparametric alternatives (Mann–Whitney U, Kruskal–Wallis). Survival analysis used Kaplan–Meier curves. Statistical processing was performed with IBM SPSS v24.

## 3. Results

### 3.1. Case Selection Flow

In 2023, the Helicopter Emergency Medical Services (HEMSs) of Castilla-La Mancha performed 2303 missions with care reports. After a critical review of the records, 322 cases were initially identified as potentially eligible. Following detailed examination, 103 patients met all inclusion criteria and were included in the final analysis. A total of 115 reports met at least one exclusion criterion; 65 of them presented issues that prevented complete or objective analysis (e.g., illegibility or poor handwriting). Another 39 reports, although fulfilling inclusion criteria, contained deficiencies that made it impossible to extract all required data.

### 3.2. Demographic Characteristics and Geographic Distribution

The cohort consisted predominantly of males (83.3%; n = 75) and females (16.7%; n = 15). The mean age was 57.77 years (standard deviation [SD] ± 18.59; range: 16–88). Patients originated mainly from the provinces of Ciudad Real (26.2%), Toledo (19.4%), and Albacete (17.5%). Monthly distribution was relatively homogeneous, with peaks in January and July (11.7% each) (Table 2 and Table 3).

### 3.3. HEMS Base Activity

Regarding operational bases, 27.2% of patients were attended by HEMS Giant 4 (Toledo), 16.5% by Giant 1 (Albacete), 31.1% by Giant 2 (Ciudad Real), and 25.2% by Giant 3 (Cuenca) (Table 4).

### 3.4. Pre-Hospital Response and Transport Times

A total of 100 valid observations were recorded for time to arrive at scene. The mean response time was 19.46 min (SD ± 11.87; range: 2–45). Air transport times were available for 39 cases, with a mean flight time of 28.90 min (SD ± 10.77; range: 11–52) (Table 5).

### 3.5. Logistical Variables and Access to the Healthcare System

The mean straight-line distance to the incident site was 57.19 km (SD ± 32.4), while mean road travel time was 53.11 min (SD ± 25.5). The straight-line distance to the receiving hospital averaged 60.0 km (SD ± 31.4), with a mean air medical transport time of 51.28 min (SD ± 23.8) (Table 5).

### 3.6. Mechanisms of Injury and Clinical Severity

The most frequent mechanism of injury was non-traumatic, medically origin cardiac arrest (49.5%), followed by traffic accidents (16.5%), falls from height (4.9%), and gastrointestinal bleeding (3.9%). A total of 38 patients were classified as severe trauma; among them, 31.6% experienced cardiac arrest. The association between severe trauma and cardiac arrest was statistically significant (χ^2^ = 29.67; *p* < 0.001) (Table 6).

### 3.7. Physiological Parameters and Clinical Scales

Initial values correspond to the first recorded assessment at the scene, while final values correspond to the last assessment before hospital arrival. The initial Shock Index (SI) was calculated for 45 patients, with a mean of 1.42 (SD ± 0.597; range: 1–3). The final SI (n = 49) showed a mean of 1.21 (SD ± 0.419; range: 0–2). The final Modified SI averaged 1.62 (SD ± 0.588; range: 0–3). The RSI index had a mean of 0.83 (SD ± 0.361; range: 0–2).

Initial ETCO_2_ (n = 29) averaged 31.69 mmHg (SD ± 16.94; range: 0–76), while final ETCO_2_ (n = 30) averaged 28.33 mmHg (SD ± 15.63; range: 0–50).

The initial Revised Trauma Score (RTS) was available for 31 patients, with a mean of 7.77 (SD = 3.57; range: 0–12). The final RTS averaged 6.84 (SD ± 3.76).

Anatomical severity scores included ISS (n = 16), with a mean of 9.88 (SD ± 11.96; range: 4–54), and NISS, with a mean of 27.56 (SD ± 13.44; range: 0–54).

Transfusion requirement was documented in 97 patients, with a mean of 0.61 units (SD ± 2.49; range: 0–17) (Table 7).

### 3.8. Mortality and Hospital Stay

Pre-hospital mortality was 21.4% (n = 22), while overall mortality was 29% (n = 45). Mean ICU stay was 9.88 days (SD ± 14.1) and mean total hospital stay was 19.7 days (SD ± 30.9). Patients who died during the pre-hospital phase had a higher initial S value.

I (mean 2.49; SD ± 1.45) compared with survivors (mean 1.89; SD ± 0.74). Among survivors, the initial RTS was 7.44 (SD ± 3.38), decreasing to 6.84 (SD ± 3.76) on final assessment.

Survival probability was estimated using the classical TRISS model (MTOS-95 coefficients). In the evaluable subgroup (n = 14), median predicted survival probability (Ps) was 0.79 (IQR 0.531–0.943). Observed survival was lower than predicted (W = −11.1 per 100 patients; Z = −1.19), with a Brier score of 0.423 and AUC of 0.292. Ps-TRISS was lowest in patients who died on scene (median 0.068), followed by in-hospital deaths (0.927 [0.651–0.983]) and in-hospital survivors (0.746 [0.593–0.863]) (Table 8).

### 3.9. Correlation Analysis and Statistical Tests

A paired-sample analysis of the Shock Index showed a decrease in the mean (1.36 to 1.25), but this was not statistically significant (t = 1.075; *p* = 0.289), with weak correlation between time points (r = 0.161; *p* = 0.320).

ROC curve analysis identified the Revised Trauma Score and SAFI index as the strongest predictors. The RTS achieved an AUC of 0.869 initially (95% CI: 0.628–1.000; *p* = 0.027) and 0.881 finally (95% CI: 0.657–1.000; *p* = 0.022). SAFI showed an AUC of 0.833 (95% CI: 0.535–1.000; *p* = 0.046). Final SI and NISS showed low AUC values (0.345 and 0.393).

Chi-square tests showed significant association only between initial SI and mortality (χ^2^ = 8.022; *p* = 0.005). A strong association was also found between severe trauma and cardiac arrest (*p* < 0.001).

**Figure A1:**
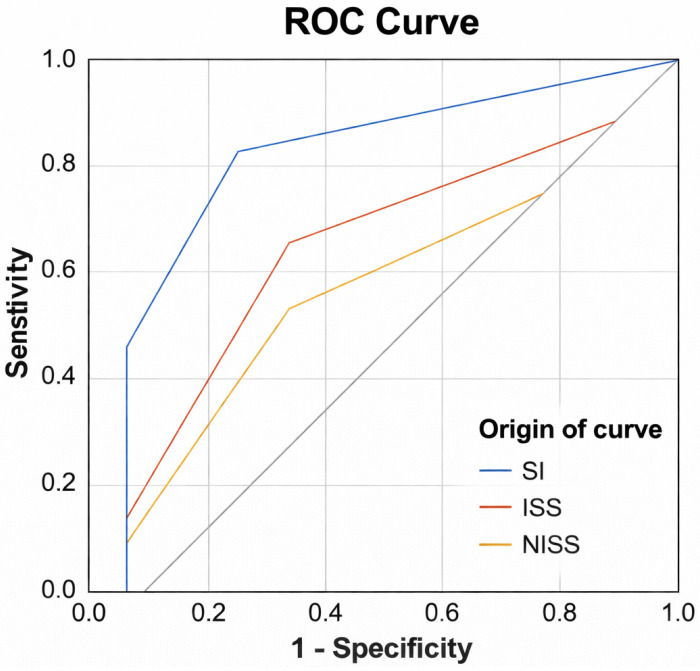
ROC curve of IS, RTS, ISS, NISS.

ANOVA identified significant differences only for SAFI (F = 14.092; *p* = 0.001). The initial Modified SI (*p* = 0.081) and Diastolic Shock Index (DSI) (*p* = 0.093) showed trends toward significance (Figure A1 and Table 9, Table 10 and Table 11)

## 4. Discussion

The present study provides relevant evidence on the feasibility of implementing REBOA in the HEMS setting in Castilla-La Mancha, based on the analysis of a cohort of 103 patients, 55.3% of whom presented cardiorespiratory arrest (CRA) during pre-hospital care. This high proportion highlights the severity of the clinical scenarios managed and aligns with findings by Treffalls et al. (2024) [29], who emphasize that the window of opportunity for applying damage-control techniques such as REBOA is critical in the first few minutes of the traumatic event, especially in patients with non-compressible bleeding.

The mean Shock Index (SI) at the start of care was 1.36 (SD = 0.38), decreasing to 1.25 (SD = 0.60) on arrival at the hospital, suggesting partial hemodynamic stabilization. This pattern is consistent with Nowadly et al. (2020) [30], who observed that REBOA can transiently improve central perfusion, allowing gaining time until definitive intervention. However, in the present study, this stabilization was achieved without the use of REBOA, reinforcing the hypothesis that its implementation could have further optimized clinical outcomes.

Overall mortality was 29%, with a pre-hospital mortality accounting for 21.4%. These values are comparable to those reported by Bini et al. (2022) [31] in the AORTA Registry, where a 34% mortality was documented in pelvic trauma patients undergoing REBOA. However, in that registry, REBOA-treated patients showed a higher survival rate compared to those who did not, suggesting that its application in this cohort may have reduced the observed mortality.

Regarding injury severity, the mean NISS was 27.56 (SD = 13.44), higher than the mean ISS of 9.88 (SD = 11.96), reflecting the presence of multiple significant injuries within the same body region rather than widespread multisystem anatomical involvement This profile corresponds with the inclusion criteria established with the inclusion criteria proposed by Ordoñez et al. (2020) [32] for the indication of REBOA in the context of damage-control resuscitation. In addition, 31.6% of severe trauma patients suffered CRA, reinforcing the need for aggressive and early interventions in this subgroup.

The literature also supports the technical feasibility of REBOA in the HEMS setting. Brede et al. (2020) [33] concluded that aortic balloon occlusion during helicopter operations is both feasible and safe, assuming appropriate personnel training and the availability of specialized equipment. Despite this, there remains a lack of consensus on the exact technical and non-technical competencies required for out-of-hospital REBOA deployment, highlighting a current gap in standardized training.

In this study, the mean times of arrival at the incident (19.46 min) and transfer to the hospital (28.90 min) provide a reasonable time window for the implementation of REBOA before hospital admission, especially in rural or difficult to access areas.

Patient selection and protocol standardization have been identified as key factors for success in REBOA implementation. The UK-REBOA trial (Jansen et al., 2023) [18,34] highlighted the critical importance of appropriate triage and structured decision-making pathways. Our study contributes to this discussion by proposing context-specific inclusion and exclusion criteria tailored to the Spanish pre-hospital system, aligned with the recommendations of the 2021 European Resuscitation Council (ERC) guidelines.

Although a formal cost-effectiveness analysis was not performed, prior literature suggests that REBOA may be more cost-effective than other advanced interventions such as ECMO (Extracorporeal Membrane Oxygenation) or massive out-of-hospital transfusion (Treffalls et al., 2024 [29]. Its ability to reduce the need for immediate surgical interventions and improve cerebral and coronary perfusion positions it as a strategic tool in the initial care of severe trauma.

Taken together, the results of this study support the clinical, logistical, and potentially economic feasibility of using REBOA in the HEMS setting and underscore the need for prospective studies and randomized clinical trials evaluating its impact on patient survival and quality of life.

The results of this study support the clinical and operational feasibility of implementing REBOA in HEMSs in Castilla-La Mancha. The high proportion of patients with severe trauma, non-compressible bleeding and refractory cardiorespiratory arrest suggests that this technique could have offered a significant benefit in terms of survival and reduced morbidity and mortality.

REBOA presents itself as a promising tool in the pre-hospital therapeutic arsenal, especially in settings with prolonged transfer times and logistical constraints. Its early application could improve the hemodynamic stability of patients, allow safer transfer and reduce the need for more invasive and costly interventions in the hospital.

The statistical results obtained reinforce the need to consider physiological parameters and established scales in decision-making. In particular, the high discriminatory capacity of the Revised Trauma Score (AUC > 0.86) and the SAFI index (AUC = 0.833) indicates that these tools are essential for identifying patients at higher risk in the HEMS setting. This finding coincides with that described by Ordoñez et al. (2020) [32] and Jansen et al. (2023) [18], who highlight the importance of integrating physiological scales into damage-control protocols.

On the other hand, the lack of significance in most simple indices (SI, ISS, NISS) suggests that their isolated use does not provide predictive value, which is consistent with recent literature (Treffalls et al., 2024) [29].

Furthermore, the significant association between initial SI and mortality (χ^2^ = 8.022; *p* = 0.005) and the differences found in SAFI (F = 14.092; *p* = 0.001) support the hypothesis that the implementation of REBOA could optimize hemodynamic stability in patients with refractory hemorrhagic shock. These data, together with previous evidence, suggest that patient selection should be based on robust physiological criteria and validated scales, prioritizing early interventions in those with the highest anatomical and functional risk.

The discrepancy between observed survival and that predicted by TRISS in this cohort suggests that the model flags patients with non-compressible hemorrhage and a high risk of rapid deterioration; this clinical profile exceeds the physiological and anatomical variables traditionally incorporated into the score. In this regard, these findings point to a window of opportunity for early implementation of advanced hemorrhage-control strategies such as REBOA, whose prospective evaluation could help optimize outcomes in patients with ostensibly favorable initial predicted survival.

When interpreting the results, the characteristics of the retrospective and observational study design, as well as the incomplete availability of some variables in the analyzed records, should be considered. Nevertheless, these findings provide relevant information within the evaluated context and may serve as a foundation for future studies with different designs to complement the evidence and support the implementation of REBOA.

Nonetheless, successful implementation requires overcoming substantial challenges, including structured personnel training, equipment standardization, and the development of clear, evidence-based clinical protocols. Ethical considerations, particularly around informed consent and the risks of pre-hospital invasive procedures, must also be addressed

## 5. Conclusions

In conclusion, REBOA represents an advanced resuscitation strategy with significant potential to improve outcomes in the pre-hospital management of patients with severe trauma and hemodynamic instability. Its integration into HEMSs should be approached through a structured, evidence-informed framework that prioritizes progressive implementation, specialized team training, and continuous evaluation of both clinical effectiveness and operational feasibility.

Future prospective studies and pilot programs are essential to validate its impact on survival, neurological outcomes, and resource utilization, particularly in rural and logistically complex settings.

## Figures and Tables

**Table 1:** Reports with Code CIE 9 URGENCIAS GUETS.

Name	Code
Unspecified Peritonitis	567.9
Traumatic amputation of leg(s), complete or partial	897
Traumatic amputation of toe	895
Traumatic amputation of foot, complete or partial	896
Traumatic amputation of arm and hand	887
Traumatic amputation of thumb, complete or partial	885
Amputations: more specific data	
Crushing of multiple and unspecified sites	929
Crushing injury of lower limb	928
Crushing injury of upper limb	927
Crushing injury of trunk and/or external genitalia	926
Unspecified complication of labor and delivery	669.9
Abdominal rigidity	789.4
Contusion of the hip	924.01
Contusion of sternum, clavicle and/or costal region	922.1
Contusion of the back and buttocks (includes lumbo-sacral-coccygeal region)	922.3
Contusion of lower limb with unspecified site	924.9
Contusion of the abdominal wall	922.2
Contusion of chest wall	922.1
Contusion of the foot	924.20
Contusion of the lower leg	924.10
Injury to heart and lung	861
Multiple contusions of lower limb	924.4
Multiple contusions on trunk	922.8
Fracture of ribs or sternum	807
Fracture of femur	821
Fracture of pelvis	808
Antepartum hemorrhage	641.9
Hemorrhage of gastrointestinal tract, unspecified	578.9
Excessive hemorrhage after abortion or ectopic or molar pregnancy	639.1
Immediate postpartum hemorrhage	666.1
Secondary and recurrent hemorrhage	958.2
Traumatic hemothorax and/or pneumothorax	860
Hemothorax, hemopneumothorax, non-traumatic hydrothorax	511.8
Wound in hip, buttock and/or thigh	890.0
Open wounds of other and unspecified parts of the trunk, except limbs	879
Chest wall injury	875
Superficial wound with opening of thoracic cavity	860.5
Multiple wounds on lower limb except thigh	894
Hypovolemia (not hypovolemic shock)	276.5
Superficial injury hip thigh, knee, leg and ankle	916
Superficial trunk injury	911
Metrorrhagia not related to menstrual cycle	626.6
Cardiorespiratory arrest	427.5
Multiple contusions	924.8
Polytraumatized	959.8
Rectorrhagia	569.3
Cardiogenic shock	785.51
Hypovolemic shock	785.59
Non-specific shock	785.50
Unspecified injury to the trunk	959.1
Unspecified injury to the hip and thigh region	959.6
Unspecified injury to the knee, leg, ankle and foot	959.7
Flail chest	807.4

**Table 2:** Sex.

		Frequency	Percentage
Sex	WOMEN	15	14.6
	MEN	75	72.8
	Total	90	87.4
Lost	System	13	12.6
Total		103	100.0

**Table 3:** Province of origin.

		Frequency	Percentage	Valid Percentage	Cumulative Percentage
Province of origin	Not specified	13	12.6	12.6	12.6
	Albacete	18	17.5	17.5	30.1
	Ciudad Real	27	26.2	26.2	56.3
	Cuenca	16	15.5	15.5	71.8
	Toledo	20	19.4	19.4	91.3
	Guadalajara	8	7.8	7.8	99.0
	Madrid	1	1.0	1.0	100.0
	Total	103	100.0	100.0	

**Table 4:** HEMS Giant.

		Frequency	Percentage	Valid Percentage	Cumulative Percentage
HEMS Giant	1	17	16.5	16.5	16.5
	2	32	31.1	31.1	47.6
	3	26	25.2	25.2	72.8
	4	28	27.2	27.2	100.0
	Total	103	100.0	100.0	

**Table 5:** Time and distances to the incident.

Parameter	N	Mean	Median	Mode	Std Dev	Min	Max	Range	Std Error	Variance
Arrival time to incident (min)	100	19.46	20.00	21.00	11.87	2.00	45.00	43		
Air travel time to hospital (min)	39	28.90	27.00	26.00	10.77	11.00	52.00	41		
Distance to incident straight line (km)	87	57.19			32.395	2	191	189	3.473	1049.443
Travel time by car (min)	87	53.11			25.459	7	157	150	2.730	648.173
Distance to hospital (km)	43	60.00			31.359	5	154	149	4.782	983.381
Travel time by car to hospital (min)	43	51.28			23.845	9	132	123	3.636	568.587

**Table 6:** Injury mechanism.

		Frequency	Percentage	Valid Percentage	Cumulative Percentage
Injury mechanism	Not specified	1	1.0	1.0	1.0
	Bicycle accident	1	1.0	1.0	1.9
	Motorcycle accident	1	1.0	1.0	2.9
	Quad bike accident	1	1.0	1.0	3.9
	Tractor accident	3	2.9	2.9	6.8
	Traffic accident	17	16.5	16.5	23.3
	Hanging	1	1.0	1.0	24.3
	Stabbing	1	1.0	1.0	25.2
	Choking	1	1.0	1.0	26.2
	Trapped	1	1.0	1.0	27.2
	Hit by a vehicle	1	1.0	1	28.2
	Fall	2	1.9	1.9	30.1
	Thoracic surgery	1	1.0	1.0	31.1
	Upper gastrointestinal bleeding	3	2.9	2.9	34
	Lower gastrointestinal bleeding	1	1.0	1.0	35
	Gunshot wound	2	1.9	1.9	36.9
	CPR	1	1.0	1.0	37.9
	Non-traumatic CPR	51	49.5	49.5	87.4
	Other non-traumatic CPR	1	1.0	1.0	88.3
	Traumatic CPR	2	1.9	1.9	90.3
	Plunge	5	4.9	4.9	95.1
	Vaginal bleeding	1	1.0	1.0	96.1
	Cardiogenic shock	1	1.0	1	97.1
	Cardiogenic shock with non-traumatic CPR	1	1.0	1.0	98.1
	Septic shock	1	1.0	1	99.0
	Esophageal varicose veins	1	1.0	1	100.0
	Total	103	100.0	100.0	

**Table 7:** Descriptive statistics of index.

Descriptive Statistics of Index	N	Mean	Standard Deviation
IS (HR/HRR) Initial	45	1.42	0.597
RSI (TAS/FC)	45	0.83	0.361
Final ETCO2	30	28.33	15.634
Initial ETCO2	29	31.69	16.937
InitiaL RTS	31	7.77	3.566
IS (FC/TAS) Final	49	1.21	0.419
Final MODIFIED IS	49	1.62	0.588
Initial REVISED TRAUMA SCORE	32	7.44	3.379
Final REVISED TRAUMA SCORE	31	6.84	3.760
ISS	16	9.88	11.960
NISS	16	27.56	13.441
Transfusion	97	0.61	2.494
Valid N (by list)	5		

**Table 8:** Pre-hospital death and pre-hospital outcome.

**Pre-Hospital Death**		**Frequency**	**Percentage**	**Valid** **Percentage**	**Cumulative Percentage**
Exitus	No	49	47.6	69.0	69.0
	Yes	22	21.4	31.0	100.0
	Total	71	68.9	100.0	
Lost data	System	32	31.1		
Total		103	100.0		
**Pre-Hospital** **Outcome**		**Average**	**N**	**Standard Deviation**	
No		1.89	10	0.738	
Yes		2.49	7	1.447	
Total		2.14	17	1.089	

**Table 9:** ROC Analysis Summary.

Variable	AUC	95% CI	*p*-Value
Revised Trauma Score (Initial)	0.869	0.628–1.000	0.027
Revised Trauma Score (Final)	0.881	0.657–1.000	0.022
SAFI Index	0.833	0.535–1.000	0.046
IS Final	0.345	0.028–0.663	NS
NISS	0.393	0.069–0.717	NS

**Table 10:** Chi-square tests.

Variable	Chi^2^	*p*-Value
SI Initial	8.022	0.005
Trauma Severity vs. CPR	29.670	<0.001

**Table 11:** ANOVA Res.

Variable	F	*p*-Value
SAFI Index	14.092	0.001
IS Modified Initial	-	0.081
DSI	-	0.093

## Data Availability

The data presented in this study are available on request from the corresponding author.

## Public Involvement Statement

No public involvement in any aspect of this research.

## Guidelines and Standards Statement

This manuscript was drafted against the STROBE for cross-sectional observational research.

## Abbreviations

The following abbreviations are used in this manuscript:

ACLS	Advanced Cardiovascular Life Support
ANOVA	Analysis of variance
AUC	Area under the curve
AIS	Abbreviated Injury Scale by trauma localization
ALS	Advanced Life Support
BLS	Basic Life Support
BPM	Beats per minute
CPR	Cardiopulmonary Resuscitation
CRA	Cardiorespiratory Arrest
DGT	Dirección General de Tráfico
Dl	Decilities
DNR	Do Not Resuscitate
DSI	Diastolic index shock
ECMO	Extracorporeal Membrane Oxygenation
ERC	European Resuscitation Council
GWCS	Glasgow Coma Scale
GUETS	Gerencia de Urgencias, Emergencias y Transporte Sanitario
HEMS	Helicopter Emergency Medical Services
ICD	International Classification of Diseases
ICU	Intensive Care Unit
ISS	Injury Severity Score
KM	Kilometers
mg	Milligrams
mmHg	Millimeters of mercury
MSI	Modified Shock Index
N	Statistical population
NISS	New Injury Severity Score
RC	Red Cross
REBOA	Resuscitative Endovascular Balloon Occlusion of the Aorta
RSI	Reserved Shock index
RTS	Revised Trauma Score
RR	Respiratory rate
SAFI	(SpO_2_/FiO_2_)x 100
SD	Continuous variables
SI	Shock Index
STO2%	Oxygen saturation
SPSS	Statistical Package for the Social Sciences
WHO	World Health Organization
